# Botanical Description of British Plants

**Published:** 1808-03

**Authors:** 


### *Botanical Description of British Plants.*

[ Continued from our last, pp. 162—168. ]

6. HIERACIUM. *H. pilosella.*

*Ang.* Creeping Mouse-ear. Mouse-ear hawk-weed.

*Gen. Desc.* Recept. generally naked. Cal. tiled, egg-shaped, sometimes double. Down mostly sitting, hair-like; rarely feathered.

*Spec. Desc.* *Stalk* leafless, flowered. *Down* simple. *Leaves* v. entire, egg-shaped, cottony underneath. *Suckers* cottony, hairy, creeping. *Cal.* hairs terminated by black globular substances. *Bloss.* red outside, pale yellow within, open at 8 A. M. close at 2 P. M. *Florets* with a broad purple stripe on the under side.—*Dry pastures, walls.* BL. May, Sept.

*Use.* This differs from the other lactescent plants, being less bitter and more astringent.—It is esteemed hurtful to sheep. An insect of the cochineal genus, (coccus polonicus,) which is much used in Poland as a substitute for the true cochineal, is often found at the roots of this plant. —*Act. Upsal,* 1752.—Goats eat it; sheep are not fond of it; horses and cows refuse it.—*Withering.*

7. HYPOCHÆRIS. *H. maculata.*

*Ang.* Spotted cat's-ears.

*Gen. Desc.* Recept. chaffy. Cal. somewhat tiled. Down on a pedicle, feathered.

*Spec. Desc.* *Stem* almost bare, with a solitary branch, and 1 or 2 strap-shaped scales. *Leaves* egg-oblong, entire, toothed, spreading on the ground, fringed, with reddish angular spots and scattered hairs. *Cal. outer scales* blackish, fringed; *inner* smooth, yellowish, hairy, composed of large scales. *Bloss.* yellow, opens at 6 A. M. closes at 4 P. M. *Seeds* wrinkled.—*Mountainous meadows, &c.* BL. July.

*Use.* The country people believe it a cure for tetters and other cutaneous eruptions; possibly through a vulgar prejudice founded on its spotted leaves.—The leaves are boiled and eaten like cabbage.—Horses are very fond of it when green, but they do not like it dry; sheep are not fond of it; cows, goats and swine eat it.—*Withering.*

8. CHICORIUM. *C. intybus.*

*Ang.* Wild succory, or chicory, or endive.

*Gen. Desc.* Recept. somewhat chaffy. Cal. double. Down about 5 teeth, indistinctly hairy.

*Spec. Desc.* *Leaves* notched. *Flowers* in pairs, sitting axillary, open at 8 A. M.; and close at 4 P. M.; fine blue individuals with 5 or 6 semi-transparent lines, a little woolly on the outside: cylinder of anthers striped blue and white. *Germen* edged with little teeth. *Summits* blue. *Seeds* oblong, four-cornered, crowned with a small greenish cup, edged with white skinny teeth. *Chaff* short, spear-shaped. *Stem* angular. *Stem-leaves* spear-shaped, embracing, toothed at the base, fringed. *Cal. outer*, 6 scales, bent back.—*Borders of cornfields*. Bl. July, Aug.

*Use.* The roots and leaves are useful aperients, acting mildly and without irritation, tending rather to abate than to increase heat, and which may therefore be safely given in hectic and inflammatory cases. Taken freely, they keep the belly open, or produce a gentle diarrrhœa; and when thus continued for some time, they have often proved salutary in beginning obstructions of the viscera, in jaundices, cachexies, hypochondriacal, and other chronical disorders.—*Lewis*. A decoction of this herb, with others of the like kind, in whey, and rendered purgative by an addition of sal. polychrest. was found a useful remedy in cases of biliary calculi, (according to Van Swieten,) and promises advantage in many complaints requiring what have been termed attenuants and resolvents. The virtues of succory reside in its milky juice; and in most plants of the order Semiflosculæ, a juice of a similar nature is to be found: what has been above said therefore of the effects of Leontodon, will in a great measure apply to this plant: the expressed juice of both, taken in large doses, frequently repeated, has been found an efficacious remedy in phthisis pulmonalis, as well as in the various affections above mentioned. The seeds, small, angular, and brown, are reckoned among the "four smaller cooling seeds."—*Woodville*. The roots are bitterer than the leaves or stalks, and these are more so than the flowers: by culture in gardens and blanching, it loses its bitterness; and, when blanched, the leaves may be eaten early in the spring in sallad. The roots also, if gathered before the stem shoots up, are eatable, and, when dried, may be made into bread.—Sheep, goats, and swine eat it; horses and cows refuse it.—*Withering*.

9. ARCTIUM. *A. lappa.*—*Lappa major. Personata. Bardana major.*

*Ang.* Burdock. Common burr. Clot-burr. Hurr-burr.

*Gen.*

*Gen. Desc.* Cal. globular; scales with hooked points, bent inwards.

*Spec. Desc.* *Leaves* heart-shaped, without thorns, on leaf-stalks, waved at the edges; *upper*, egg-spear-shaped. *Heads* slightly woolly. *Stems* reddish, with soft white bristles. *Fruit-st.* axillary. *Cal. scales*, green and fleshy at the base, purple towards the top, keeled, ending in long stiff awns, yellow at the hooked ends. *Bloss.* tube white, border red. *Anth.* purple. *Style* white.—*Road sides, rubbish.* Bl. July, August.

*Use.* The Pharmacopœias direct the root for medical use: it has no smell, but tastes sweetish, mixed with a slight bitterishness and roughness. Bergius states its virtue to be mundificans, diuretica, diaphoretica; and many instances are upon record in which it has been successfully employed in a great variety of chronic diseases, as scurvy, rheumatism, gout, lues venerea, and pulmonic complaints. Henry III. of France, was cured of a venereal taint by a decoction of its roots. (*See Reverius, Obs.* 41.) As a diuretic, it has been known to succeed in dropsical cases, where other powerful medicines had been ineffectually used; and as it neither excites nausea, nor increases irritation, it may deserve a trial where more active remedies are improper. The seeds also possess a diuretic quality, and have been given with advantage in the dose of a drachm, in calculous and nephritic complaints; and in the form of emulsion, as a pectoral. The root is generally used in decoction, which may be made by boiling 2 oz. of the fresh root in 3 pints of water to 2, which, when intended as a diuretic, should be taken in the course of two days, or if possible, in four and twenty hours.—*Woodville.* Boerhaave recommends a decoction of the root in pleurisies, peripneumonies, and malignant fevers: it is said to have cured the venereal disease. An elixir of it has been much extolled in the gout, and an emulsion of the seeds has a powerful diuretic quality:—outwardly applied, the leaves are useful in head-achs, gout, and œdematous swellings.—The roots and stalks are esculent and nutritive; the stalks, for this purpose, should be cut before the plant flowers, the rind peeled off, and then boiled and served in the manner of cardoons, or eaten raw as a sallad, with oil and vinegar.—*Lightfoot.* A decoction of the root is esteemed by some very sensible physicians, as equal, if not superior to that of sarsaparilla. The stems gathered before the flowers appear, and stripped of the rind, are boiled and eaten like asparagus. Boys catch bats, by throwing the prickly heads up into the air. Cows and goats eat it; swine are not fond of it; horses refuse it.—*Withering.*

10. Serratula. *S. tinctoria.*

*Ang.* Common saw-wort.

*Gen. Desc.* Cal. nearly cylindrical, tiled; scales not awned.

*Spec. Desc.* *Leaves* lyre-shaped and wing cleft, fringed, terminating segment very large; wings spear-shaped, sharply serrated, woolly; sometimes entire, alternate, half embracing. *Florets* all alike. *Flowers* simple, or in clusters, purple. *Down* yellowish, shining, hairy. *Recept.* bristly. *Stem* 2 or 3 feet high, firm, four-cornered, scored, smooth.—*Woods, pastures.* Bl. July.

*Var.* with white blossoms; and another with jagged leaves.

*Use.* This plant is very much used by dyers, to give a yellow colour; but, as it is inferior to the Reseda, its use is confined to the coarser woollen cloths.—Goats eat it; horses are not fond of it; cows, sheep, and swine refuse it.—*Withering.*

11. Serratula. *S. arvensis.*—*Carduus arvensis.*

*Ang.* Corn saw-wort. Way-thistle.

*Gen. Desc.* *As above.*

*Spec. Desc.* *Leaves* toothed, thorny. *Down* of the seed very long, hair like. *Bloss.* pale purple. It has the habit of a Carduus.—*Corn fields, road sides.* Bl. July.

*Use.* It is said to yield a very pure vegetable alkali, when burnt.—Goats eat it; neither cows, horses, sheep, nor swine are fond of it,—*Linn.*; but horses sometimes eat the young tops.—*Withering.*

12. Carduus. *C. lanceolatus.*

*Ang.* Spear thistle.

*Gen. Desc.* Calyx bellying, tiled. Scales thorny. Receptacle hairy.

*Spec. Desc.* *Leaves* decurrent, with winged clefts, hispid, segments stradling; half embracing, underneath cottony and sea-green, above hairy and deep green. *Stem* angular, cottony, purplish, 2 to 5 feet high. *Calyxes* egg-shaped, thorny, woolly dry, innermost scales without thorns; numerous ranges of spear-shaped scales, ending in stiff white thorns. *Bloss.* purple. *Down* feathered.—*Rubbish, waste places.* Bl. July August.

*Use.* Few plants are more disregarded than this, and yet its use is very considerable. If a heap of clay is thrown up, nothing would grow upon it for several years, did did not the seeds of this plant, wafted by the wind, fix and vegetate thereon: under the shelter of this, other vegetables appear, and the whole soon becomes fertile. The flowers, like those of the artichoke, have the property of curdling milk. Sheep and swine refuse it; neither horses, cows, nor goats are fond of it.—*Withering.*

Almost all the species of this genus may be eaten like the burdock, before the flowers are formed.—*Ib.*

13. Carduus. *C. Marianus.*

*Ang.* Milk thistle. Lady's thistle.

*Gen. Desc.* As above.

*Spec. Desc.* Leaves sitting, halberd shaped, and wing-cleft, embracing, thorny, with broad white veins. *Cal.* without any leaves near it, set with strong thorns an inch long, channelled and set with other little thorns. *Bloss.* large, purple. *Ditch banks, borders of corn fields, rubbish.* Bl. August.

*Use.* When young this is eaten as a sallad. The young stalks peeled, and soaked in water, to take off the bitterness, are excellent. The scales of the cup are as good as artichokes; the root is good to eat early in the spring.—*Withering.*

14. Onopordon. *O. acanthium.*

*Ang.* Common argentine. Cotton thistle.

*Gen. Desc.* Recept. like a honeycomb. Calyx bellying. Scales sharp-pointed.

*Spec. Desc.* *Cal. scales* expanding, points standing out, awl-shaped. *Leaves* egg-oblong, indented; *lower* extremely large, with deep triangular teeth, again toothed, each ending in a sharp whitish thorn. *Stem* with a leafy border, thorny. *Plant* hoary green, cottony. *Heads,* single, terminating, upright. *Bloss.* sometimes white. *On rubbissh and road sides.* Bl. July.

*Use.* The receptacle and the young stems, may be boiled and eaten like the artichoke. The ancients thought this plant a specific in cancerous cases. Horses, cows, and sheep refuse it.— *Withering.*

Carlina. *C. vulgaris.*

*Ang.* Wild carline thistle.

*Gen. Desc.* Calyx radiated; scales next the blossoms long, coloured.

*Spec. Desc.* *Stem* 12 to 15 inches high, cylind. ribbed, purple, with many flowers in a corymbus, terminating. *Rays of cal.* yellow white. *Leaves* numerous, covering the stem, decreasing in size upwards, *lower* sitting, *upper*

embracing. *Bloss.* tube whitish, border of the outer flower purple, of the *inner* whitish. *Seed,* woolly. *Down* pale brown, sitting, thrice as long as the seed. *Dry meadows, and pastures.* Bl. June.

*Use.* The root was once kept in the shops as an *alexipharmic* and *astringent,* but is out of use.—*Hill.* This is said to be an excellent remedy in *hysterical* cases. *See Amœn. Acad. iii, p.* 64. The flowers expand in dry, and close in moist weather; and, as they retain this property for a long time, they are often employed as hygrometers. Is presence indicates a very barren soil. Goats eat it, cows refuse it.—*Withering.*

16. Eupatorium. *E. cannabinum.*

*Ang.* Hemp agrimony, Dutch agrimony. Water agrimony. Water hemp. Common hemp weed.

*Gen. Desc.* Recept. naked. Down feathered. *Cal.* oblong, tiled. Style long, cloven half way down.

*Spec. Desc.* *Cal.* 5-flowered, skinny, colored, hairyish, scales few, strap-shaped. *Leaves* with finger-like divisions, slightly woolly. *Leafits* 3 to 5, serrated at the base, towards the point entire. *Stem* 3 to 4 feet high, branched, reddish, woolly. *Seeds* black, scored, smooth. *Down* sitting, hair-like. *Flor.* 5 and 6. *Bloss.* purplish red, clefts shallow. *Styles* and *summits* with a tinge of red. *Banks of rivers and brooks.* Bl. July, August.

*Use.* This plant has a bitter taste. A decoction of the root operates as a violent emetic and cathartic; it is sometimes taken by the lower classes in jaundice, dropsy, and cachexies, but it is a rough medicine, and must be used with caution. Boerhaave used an infusion of this plant to foment ulcers and putrid sores. It is used by the Turks, says Tournefort, to cure the scurvy. An ounce of the juice, or a drachm of the extract, is a dose.—*Lightfoot.* An infusion of a handful of it vomits and purges smartly. An ounce of the root in decoction is a full dose. In smaller doses it is taken by the Dutch peasants as an alterative, and as an antiscorbutic. Goats eat it; horses, cows, sheep, and swine refuse it.—*Withering.*

( To be continued. )

